# Divide-and-conquer routing for learning heterogeneous individualized capsules

**DOI:** 10.1371/journal.pone.0329202

**Published:** 2025-07-30

**Authors:** Hailei Yuan, Qiang Ren

**Affiliations:** 1 School of Yonyou Digital and Intelligence, Nantong Institute of Technology, Nantong, China; 2 School of Computer Science and Technology, Tongji University, Shanghai, China; New York University Abu Dhabi, UNITED ARAB EMIRATES

## Abstract

Capsule Networks (CapsNets) have demonstrated an enhanced ability to capture spatial relationships and preserve hierarchical feature representations compared to Convolutional Neural Networks (CNNs). However, the dynamic routing mechanism in CapsNets introduces substantial computational costs and limits scalability. In this paper, we propose a divide-and-conquer routing algorithm that groups primary capsules, enabling the model to leverage independent feature subspaces for more precise and efficient feature learning. By partitioning the primary capsules, the initialization of coupling coefficients is aligned with the hierarchical structure of the capsules, addressing the limitations of existing initialization strategies that either disrupt feature aggregation or lead to excessively small activation values. Additionally, the grouped routing mechanism simplifies the iterative process, reducing computational overhead and improving scalability. Extensive experiments on benchmark image classification datasets demonstrate that our approach consistently outperforms the original dynamic routing algorithm as well as other state-of-the-art routing strategies, resulting in improved feature learning and classification accuracy. Our code is available at: https://github.com/rqfzpy/DC-CapsNet.

## 1 Introduction

In recent years, deep learning has driven remarkable progress in computer vision, achieving significant success in image classification [1–8], object detection [9–15], and image segmentation [16, 17]. Convolutional neural networks (CNNs), one of the most widely adopted deep learning architectures, leverage local receptive fields, weight sharing, and hierarchical feature extraction to achieve outstanding performance. By extracting features through convolutional layers, CNNs enable robust recognition by detecting object components and their presence. Despite their effectiveness, CNNs exhibit inherent limitations in capturing spatial relationships between object components. This issue arises from their reliance on translation-invariant convolutional operations, which do not explicitly encode spatial dependencies. Additionally, commonly used max-pooling operations discard essential positional and structural information [4], disrupting the fine-grained representation of objects. While pooling contributes to viewpoint invariance [1], it simultaneously weakens the network’s ability to model complex transformations such as object deformations, rotations, and part-whole relationships.

To address these limitations, capsule networks (CapsNets) have been introduced as an alternative to conventional CNNs, offering improved capabilities in capturing and representing features across different viewpoints. Unlike traditional CNNs, which rely on scalar neurons, CapsNets represent entities using vector capsules, where each capsule encodes not only the presence of a feature but also its instantiation parameters, such as pose, scale, orientation, and deformation [18, 19]. This structured representation enables CapsNets to preserve spatial hierarchies more effectively. Instead of max-pooling, CapsNets employ a dynamic routing mechanism, which iteratively refines the coupling coefficients between lower-level and higher-level capsules. This mechanism ensures that feature information is selectively transmitted based on agreement, allowing the network to learn meaningful spatial relationships and enhancing its robustness to affine transformations and occlusions [20].

Despite these advantages, CapsNets still face several limitations. First, the transformation of scalar neurons into vector capsules via convolutional capsule layers necessitates a fully connected routing mechanism, where iterative dynamic routing globally adjusts coupling coefficients across all capsules. This process introduces substantial computational overhead [19], significantly increasing training time and limiting scalability. Although protocol-based routing mechanisms have been explored to mitigate these challenges [19, 21], they inherently introduce excessive parameters and higher computational complexity compared to traditional approaches such as pooling [1, 2]. Additionally, the initialization of routing coefficients remains a critical issue. When initialized based on the number of high-level capsules, the bottom-up feature aggregation process is disrupted, contradicting the hierarchical nature of capsule transformations. Conversely, initializing coefficients based on low-level capsules often leads to exceedingly small initial values, causing excessive smoothing effects that hinder feature discrimination [22, 23].

To address these challenges, this study introduces a divide-and-conquer routing algorithm to enhance CapsNet by structuring the primary capsules into groups and leveraging independent feature subspaces for more refined feature learning. This approach improves the model’s ability to capture diverse local structures while simultaneously reducing computational complexity. By partitioning the primary capsules into distinct groups, the initialization of capsule coupling coefficients can be computed based on the number of lower-level capsules, aligning more naturally with the hierarchical nature of capsule-based feature aggregation. This results in a more structured and semantically meaningful composition of features. Moreover, replacing the fully connected dynamic routing mechanism with a grouped routing strategy simplifies the iterative routing process, significantly reducing the computational burden. By introducing this structured routing paradigm, our method achieves a more efficient and scalable capsule-based representation, making it particularly well-suited for complex visual recognition tasks.

However, it is important to recognize that while the proposed method offers clear advantages in terms of efficiency and local feature discrimination, it may not consistently outperform the original dynamic routing algorithm in scenarios that require modeling global spatial relationships and transformation invariance. For instance, on datasets such as SMALLNORB [48] and AFFNIST [49], which are specifically designed to assess robustness to spatial transformations, our approach performs less favorably compared to standard dynamic routing, with a particularly noticeable accuracy gap on SMALLNORB. This outcome reveals an inherent trade-off in the divide-and-conquer strategy: by emphasizing localized feature subspaces through grouping, the model may lose the global coherence necessary to capture holistic pose and viewpoint variations. As a result, while the proposed strategy is particularly well-suited for tasks that benefit from fine-grained local feature representations, such as medical image analysis or standard object classification, it may be less effective in domains where comprehensive spatial reasoning is essential.

To comprehensively evaluate the effectiveness of the proposed method, we conduct extensive experiments on multiple benchmark image classification datasets. The results demonstrate that our approach consistently outperforms the original dynamic routing algorithm in terms of accuracy and robustness. Moreover, when integrated with several state-of-the-art capsule network routing strategies, our proposed divide-and-conquer routing framework leads to further performance improvements across various datasets. In addition, we perform ablation studies to assess the influence of key architectural components, such as capsule grouping configurations and the number of routing iterations. These analyses confirm the individual and combined contributions of our design choices to the overall performance. To provide further insight into the representational power of the model, we visualize the feature activations of different capsule groups. The visualizations reveal that our method enables more diverse and semantically meaningful part-level representations, focusing on salient local structures that contribute to improved image recognition accuracy.

In summary, our main contributions are as follows:

1. The conventional initialization of coupling coefficients follows a top-down paradigm, which contradicts the hierarchical compositional nature of capsules. In contrast, bottom-up initialization tends to produce extremely small coefficients, leading to over-smoothing effects. To address this, we propose a divide-and-conquer routing algorithm that partitions lower-level capsules into groups, mitigating the issue of excessively small initialization values and improving the stability of coefficient updates.
2. By introducing intra-group routing, we significantly reduce the computational burden associated with fully connected dynamic routing. Simultaneously, inter-group routing facilitates information exchange between capsule groups, ensuring that feature interactions across different groups are preserved. This structured approach not only enhances model performance but also improves computational efficiency.
3. Extensive experiments on multiple benchmark datasets demonstrate that our method effectively improves upon both the original dynamic routing algorithm and other advanced routing strategies. Furthermore, visualization of different capsule groups provides insights into how capsules capture category-specific local features, offering a more interpretable understanding of the network’s internal feature representations.

## 2 Related work

### 2.1 Capsule networks

CapsNets [18] introduce a novel paradigm for entity representation using capsules. Unlike traditional scalar outputs, capsules are represented as vectors, where each element encodes a distinct characteristic of the entity, and the magnitude of the vector reflects the confidence in the entity’s existence. High-level capsules represent the output classes of the network, enabling more robust representations.

In a standard CapsNet architecture, raw input features are first mapped to low-level capsules, which are then routed to higher-level capsules for further abstraction. Specifically, in image classification tasks, the network begins with convolutional layers that convert pixel intensities into lower-level visual capsules. These capsules are subsequently routed to higher-level capsules, which capture more abstract visual representations. A CapsNet typically consists of multiple capsule layers, each incorporating its own routing procedure.

Consider a low-level capsule, denoted as ${𝐮}_{i}$, from the *L*-th layer, which contains *N* capsules. Let ${𝐬}_{j}$ represent a high-level capsule from the (L+1)-th layer, which contains *M* capsules. The routing mechanism between layers is governed by the following formulation:

$${\hat{𝐮}}_{j|i}={𝐖}_{ij}{𝐮}_{i},$$ (1)

$${𝐬}_{j}=\sum_{i=1}^{N}c_{ij}{\hat{𝐮}}_{j|i}.$$ (2)

Here, ${𝐖}_{ij}$ is the transformation matrix that maps the low-level capsule ${𝐮}_{i}$ to its predicted high-level counterpart ${\hat{𝐮}}_{j|i}$. The coupling coefficient *c*_*ij*_ quantifies the degree to which the predicted vector ${\hat{𝐮}}_{j|i}$ from the low-level capsule contributes to the activation of the high-level capsule ${𝐬}_{j}$.

To ensure that the output of each high-level capsule ${𝐬}_{j}$ remains within a normalized range, it is passed through a non-linear squashing function g(·):

$${𝐯}_{j}=g({𝐬}_{j})=\frac{||{𝐬}_{j}|{|}^{2}}{1+||{𝐬}_{j}|{|}^{2}}\frac{{𝐬}_{j}}{\left||{𝐬}_{j}|\right|}.$$ (3)

The coupling coefficients *c*_*ij*_ are iteratively refined during the dynamic routing process. At each iteration, the logit values *b*_*ij*_ are updated to reflect the agreement between the predicted capsule output ${\hat{𝐮}}_{j|i}$ from the lower layer and the current estimate of the higher-layer capsule ${𝐯}_{j}$, based on their scalar product ${\hat{𝐮}}_{j|i}\;\cdot \;{𝐯}_{j}$. The coupling coefficients are then computed using a softmax function over all higher-layer capsules:

$$c_{ij}=\frac{\exp(b_{ij})}{\sum_{k=1}^{M}\exp(b_{ik})}.$$ (4)

Initially, all logits *b*_*ij*_ are set to zero and are incrementally adjusted during each iteration according to the routing updates. The normalization ensures that $\sum_{j=1}^{M}c_{ij}=1$, constraining the contribution of each lower-layer capsule across all potential parent capsules. The entire routing process is typically repeated for *r* iterations, during which both the coupling coefficients *c*_*ij*_ and the capsule activations ${𝐬}_{j}$ are iteratively refined.

By representing information in vector form and employing dynamic routing, CapsNets significantly improve the representation of complex visual data. This architecture addresses the limitations of conventional CNNs by enhancing robustness to spatial transformations and enabling more sophisticated feature learning. The dynamic routing algorithm is presented in Algorithm 1.

**Algorithm 1 Dynamic routing algorithm for capsule networks.**

**Require:** Input capsules ${𝐮}_{i}$, number of iterations *r*

**Ensure:** Output capsules ${𝐯}_{j}$

1: Initialize logits $b_{ij}\leftarrow 0$ for all capsule pairs (*i*,*j*)

2: **for** each iteration *t* = 1 to *r* **do**

3:   Compute coupling coefficients using Eq 4: $c_{ij}=\frac{\exp(b_{ij})}{\sum_{k=1}^{M}\exp(b_{ik})}$

4:   Compute weighted sum of capsule outputs using Eq 2: ${𝐬}_{j}=\sum_{i=1}^{N}c_{ij}{\hat{𝐮}}_{j|i}$

5:   Apply squashing function from Eq 3: ${𝐯}_{j}=g({𝐬}_{j})=$ $\frac{\|{𝐬}_{j}{\|}^{2}}{1+\|{𝐬}_{j}{\|}^{2}}\frac{{𝐬}_{j}}{\left\|{𝐬}_{j}\right\|}$

6:   Update logits: $b_{ij}\leftarrow b_{ij}+{\hat{𝐮}}_{j|i}\cdot {𝐯}_{j}$

7: **end for**

8: **return** ${𝐯}_{j}$

### 2.2 Efficiency and scalability in capsule networks

CapsNets [18] have demonstrated effective capabilities in representing complex entities through dynamic routing, but their iterative nature incurs significant computational costs, limiting scalability. To address these issues, several optimizations have been proposed. One key improvement involves matrix capsules, which utilize the Expectation-Maximization (EM) algorithm to refine feature aggregation, leading to robust classification, especially in small datasets [19]. Additionally, the VBRouting method [21] applies variational Bayesian techniques to model a mixture of Gaussians, enforcing sparsity and enhancing capsule flexibility. Various studies have focused on reducing computational overhead through alternative routing strategies. Optimized dynamic routing approaches [24] improve initialization and update strategies for coupling coefficients, accelerating convergence. Sparse CapsNet models [25] reduce redundancy in the routing process, while kernel density estimation-inspired routing methods [26] speed up convergence without sacrificing accuracy. Other advances include AT-CapsNet [27], which replaces vector length computation with single-layer perceptrons to better leverage length and direction information, and cognitive consistency-based routing algorithms [28] that align capsule operations with human brain functions.

In addition to dynamic routing improvements, structural modifications have been explored to reduce parameter complexity. Techniques such as capsule grouping [29, 30] share transformation weights among groups to lower routing parameter counts. GRMR-CapsNet adapts between group routing and max-pooling to ensure that only the most informative features are passed to higher-level capsules. GF-CapsNet integrates a distance network for metric-based capsule activation, enhancing interpretability and reducing overhead. AR-CapsNet [31] and GR-CapsNet [32] further enhance feature extraction by leveraging attention mechanisms to improve routing efficiency and spatial relationships. Lightweight architectures also aim to reduce complexity while maintaining expressive power. DeepFeat-Caps [33] uses smaller convolutional kernels, and PT-CapsNet [34] balances efficiency with expressiveness through the selective reduction of redundant computations. Similarly, 1D-ConvCapsNet [35] employs one-dimensional convolutions to minimize spatial and computational overhead, especially in sequential data tasks. Additionally, methods like AA-Caps [36] and IAR-CapsNet [37] introduce self-routing and non-iterative mechanisms to reduce the complexity of CapsNets while preserving the network’s representational power. Further efficiency gains come from methods that integrate lightweight convolutional operations with structural modifications. MobileCapsNet [38] replaces standard convolutions with depthwise separable convolutions, reducing the parameter count. DPDH-CapNet [39] introduces a dual-path dynamic hashing mechanism to selectively activate relevant capsule pathways, improving efficiency. Other methods, like L-CapsNet [40], use pruning strategies to eliminate redundant capsules, and ResCaps [41] optimizes routing connectivity, reducing memory usage and processing time. Additionally, RS-CapsNet [42] introduces a robust routing mechanism to improve convergence speed and model stability.

While existing methods have made significant advancements in enhancing the efficiency of CapsNets, they often focus either on global routing improvements or on introducing local strategies. However, these approaches frequently overlook the trade-off between global agreement calculations and localized feature learning, resulting in inefficiencies in capturing fine-grained representations. To address this limitation, we propose a divide-and-conquer strategy that restructures dynamic routing to emphasize localized feature extraction while preserving the integrity of global capsule interactions. By selectively partitioning routing operations and prioritizing essential features at different levels of abstraction, our method enhances CapsNet’s ability to learn hierarchical representations. The following section details our proposed approach, highlighting how it improves dynamic routing efficiency and strengthens feature extraction in a computationally efficient manner.

## 3 Materials and methods

In this section, we first introduce the framework for multi-type feature learning based on a divide-and-conquer strategy. Next, we provide a detailed explanation of how the divide-and-conquer approach is implemented in our method. Finally, we describe the loss function employed to optimize the training process.

### 3.1 Overall framework

**Fig 1:**
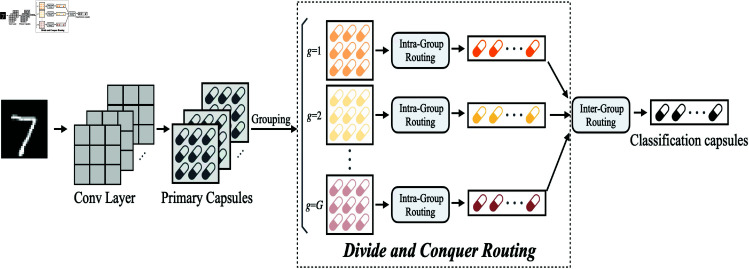
The architecture of DC-CapsNet consists of four main components: a convolutional layer, a primary capsule layer, a divide-and-conquer module, and inter-group routing. The convolutional layer extracts low-level features from the input image, which are then processed by the primary capsule layer to capture spatial hierarchies and pose variations. The divide-and-conquer module partitions the fully connected dynamic routing process into multiple groups, each focusing on learning distinct local vector representations associated with different classes. By structuring the routing process into independent groups, computational cost is reduced while preserving model accuracy and expressiveness. Finally, inter-group routing aggregates the locally learned capsule representations for final training.

We propose the Divide-and-Conquer Dynamic Routing Capsule Network (DC-CapsNet) to enhance the efficiency of dynamic routing while preserving CapsNets’ ability to capture fine-grained local features relevant to classification. As illustrated in Fig 1, the architecture of DC-CapsNet consists of four key components: a convolutional layer, a primary capsule layer, a divide-and-conquer module, and inter-group routing. The convolutional layer extracts low-level features from the input image, which are subsequently processed by the primary capsule layer to encode spatial hierarchies and pose variations. The divide-and-conquer module partitions the fully connected dynamic routing process into multiple groups, each specializing in learning distinct local vector representations associated with different class-specific characteristics. By decomposing the routing process into independent groups, DC-CapsNet effectively reduces computational overhead while maintaining representational capacity and classification performance. Finally, inter-group routing aggregates locally learned capsule representations to facilitate the final training process.

### 3.2 Coefficient reverse initialization

**Fig 2:**
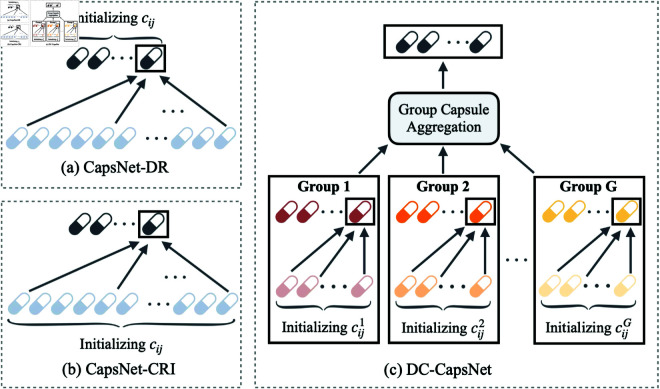
Illustration of coupling coefficient initialization and capsule aggregation methods. (a) CapsNet-DR: Standard dynamic routing with coupling coefficients initialized using softmax over upper capsules, leading to normalization across classes. (b) CapsNet-CRI: Reverse initialization with softmax over lower capsules, resulting in normalization across input capsules. (c) DC-CapsNet (Ours): Group-based reverse initialization and aggregation, aligning coefficient normalization with the hierarchical structure and avoiding the over-smoothing of coupling coefficients, which limits capsule activation in CapsNet-CRI.

In the original dynamic routing algorithm, the coupling coefficient *c*_*ij*_ between a lower-layer capsule *i* and an upper-layer capsule *j* is initialized by applying a softmax over all upper capsules, such that $\sum_{j}c_{ij}=1$. While this yields uniform initial probabilities across upper capsules, it disregards the number of lower-layer capsules contributing to each upper capsule. Ideally, the initialization should reflect the aggregation structure defined by routing Eq 2, where each upper capsule ${𝐬}_{j}$ is computed as a weighted sum over numerous lower capsules. This process is illustrated in Fig 2 (a), where CapsNet-DR performs standard dynamic routing with normalization across classes.

This mismatch in normalization direction introduces an inconsistency: the coefficients are normalized across upper capsules (typically corresponding to the number of classes), whereas aggregation takes place over a much larger set of lower-layer capsules. In light of this observation, we explore a Coefficient Reverse Initialization (CRI) scheme, which applies softmax over lower capsules for each upper capsule, thereby aligning the normalization with the actual aggregation structure. As shown in Fig 2 (b), this reverse initialization results in coefficient normalization across input capsules. In most cases, the number of lower-layer capsules far exceeds the number of classes, leading to extremely small initial *c*_*ij*_ values. Consequently, the contribution of each lower capsule is diminished in early iterations, resulting in an overly smooth routing process that impedes the model’s ability to learn distinct and discriminative part-whole relationships.

However, as shown in Table 1, applying this modification alone (CapsNet-CRI) yields degraded performance in the fully connected routing setting. We attribute this to the absence of architectural constraints: without structural regularization, reverse initialization can produce unstable or excessively sparse coupling coefficients that lack semantic focus.

**Table 1:** Experimental results comparing the classification accuracy (Acc., %) and standard deviation (Std.) of the proposed method with the original dynamic routing algorithm. CNN refers to a convolutional neural network with a parameter count comparable to the original dynamic routing capsule network. CapsNet-DR refers to the capsule network using the standard dynamic routing algorithm, CapsNet-CRI denotes the dynamic routing algorithm with inverse initialization of the coupling coefficients, DC-CapsNet-DR applies the group strategy with standard dynamic routing, and DC-CapsNet (Ours) is our full method.

Model	Metric	MNIST	F-MNIST	K-MNIST	SVHN	CIFAR-10	SMALLNORB	AFFNIST	IDRID	ISIC
CNN	Acc.	99.44	92.98	97.48	88.97	70.71	82.00	99.20	55.34	64.33
	Std.	±0.00030	±0.00070	±0.00040	±0.00055	±0.00320	±0.00036	±0.00030	±0.00025	±0.00500
CapsNet-DR	Acc.	99.65	93.02	98.30	93.65	76.05	**89.70**	**99.66**	62.14	63.33
	Std.	±0.00018	±0.00044	±0.00024	±0.00033	±0.00192	±0.00021	±0.00020	±0.00015	±0.00300
CapsNet-CRI	Acc.	99.61	92.45	97.44	91.94	72.82	82.71	99.51	58.42	59.27
	Std.	±0.00024	±0.00058	±0.00032	±0.00044	±0.00256	±0.00028	±0.00027	±0.00020	±0.00400
DC-CapsNet-DR	Acc.	99.66	93.09	98.31	93.74	76.13	88.21	99.58	63.21	64.82
	Std.	±0.00015	±0.00032	±0.00020	±0.00025	±0.00145	±0.00018	±0.00016	±0.00012	±0.00230
DC-CapsNet (Ours)	Acc.	**99.67**	**93.13**	**98.32**	**93.81**	**76.17**	87.49	99.59	**64.75**	**65.45**
	Std.	±0.00012	±0.00029	±0.00016	±0.00022	±0.00128	±0.00015	±0.00014	±0.00010	±0.00200

This observation suggests that effective routing requires not only a better initialization scheme but also an architectural framework that constrains the routing space to maintain meaningful feature groupings. To this end, we build upon the divide-and-conquer strategy by partitioning the capsule routing process into smaller, semantically coherent groups. As depicted in Fig 2 (c), our proposed DC-CapsNet adopts a group-based reverse initialization and aggregation mechanism, which aligns the coefficient normalization with the hierarchical structure of the capsule network. This design improves both the stability and efficiency of routing while providing the structural foundation necessary for reverse initialization to function effectively. The details of this strategy are presented in the following section.

### 3.3 Divide-and-conquer dynamic routing

To address the challenge of excessively small coupling coefficients and enhance the efficiency of dynamic routing, we propose a divide-and-conquer strategy that partitions the fully connected dynamic routing process into multiple groups. Each group focuses on learning distinct types of vector features, thereby reducing computational complexity while preserving feature diversity.

To further optimize the routing process, we introduce a logically consistent Coefficient Reverse Initialization strategy to update the coupling coefficients. Specifically, for each group, the coupling coefficient $c_{ij}^{g}$ of each capsule is computed iteratively. This ensures effective aggregation of capsules corresponding to the same feature type, thereby preserving the unique characteristics of each feature. The coupling coefficients are updated using the following softmax function:

$$c_{ij}^{g}=\frac{\exp(b_{ij}^{g})}{\sum_{k=1}^{\frac{N}{G}}\exp(b_{kj}^{g})},$$ (5)

where $b_{ij}^{g}$ is the initial parameter that encodes the consistency between the lower-level and higher-level capsules within group *g*. The number of groups *G* is determined by partitioning the total number of capsules *N* into smaller, more manageable subsets, typically based on the complexity or dimensionality of the features. By partitioning the routing process into independent groups g∈{1,2,…,G}, our method reduces computational overhead and accelerates convergence while preserving the representational capacity of the model. This group-based approach allows for more efficient routing, particularly in datasets with high variability, ensuring that the model can learn discriminative features effectively.

As shown in Fig 1, for the MNIST dataset, we partition the convolutionally processed capsules into 32 groups, with each group containing 32 lower-level capsules. Within each group, we apply Coefficient Reverse Initialization, setting the initial value of each coupling coefficient $c_{ij}^{g}$ to approximately 0.028. This value is significantly higher than the original initialization of 0.0008, preventing overly smooth capsule aggregation and ensuring the contribution of each lower-level capsule to the higher-level capsules.

Following this, intra-group routing is performed. For an input capsule representation ${𝐮}_{i}^{g}$ belonging to group *g*, we first compute the prediction vectors ${\hat{𝐮}}_{j|i}^{g}$ by multiplying the input capsule with the corresponding weight matrix. During each iteration of intra-group routing, the coupling coefficients are computed using Eq 5.

After obtaining the coupling coefficients, we compute the weighted sum of prediction vectors for each high-level capsule:

$${𝐬}_{j}^{g}=\sum_{i\in g}c_{ij}^{g}{\hat{𝐮}}_{j|i}^{g},$$ (6)

where $c_{ij}^{g}$ denotes the coupling coefficient for the *i*-th capsule within group *g*.

Next, we apply the squashing function to ensure that the length of the output vector represents the probability of the entity’s existence:

$${𝐯}_{j}^{g}=\frac{||{𝐬}_{j}^{g}|{|}^{2}}{1+||{𝐬}_{j}^{g}|{|}^{2}}\frac{{𝐬}_{j}^{g}}{\left||{𝐬}_{j}^{g}|\right|}.$$ (7)

Finally, we update the logits based on the agreement between the prediction vector and the output capsule:

$$b_{ij}^{g}=b_{ij}^{g}+{\hat{𝐮}}_{j|i}^{g}\cdot {𝐯}_{j}^{g}.$$ (8)

This iterative process refines the coupling coefficients, ensuring that capsules corresponding to the same feature type are effectively aggregated.

Next, inter-group routing is applied to aggregate the upper-layer capsules obtained from all groups, yielding the final discriminative capsules. This process ensures the effective combination of learned features from all groups, resulting in the most informative capsule representation for classification. The coupling coefficients between groups, denoted as $c_{gj}^{\prime }$, are iteratively updated to refine the aggregation of capsules across groups. These coupling coefficients are computed using the following softmax function:

$$c_{gj}^{\prime }=\frac{\exp(b_{gj}^{\prime })}{\sum_{k=1}^{G}\exp(b_{kj}^{\prime })},$$ (9)

where $b_{gj}^{\prime }$ is the initial parameter that encodes the consistency between capsules in group *g* and capsule *j*.

After computing the inter-group coupling coefficients, we calculate the weighted sum of the capsules from all groups:

$${𝐬}_{j}^{final}=\sum_{g=1}^{G}c_{gj}^{\prime }{𝐯}_{j}^{g},$$ (10)

where $c_{gj}^{\prime }$ represents the updated coupling coefficient for the routing between group *g* and capsule *j*, and ${𝐯}_{j}^{g}$ is the output capsule from group *g*.

We then apply the squashing function to normalize the resulting vector:

$${𝐯}_{j}^{final}=\frac{||{𝐬}_{j}^{final}|{|}^{2}}{1+||{𝐬}_{j}^{final}|{|}^{2}}\frac{{𝐬}_{j}^{final}}{\left||{𝐬}_{j}^{final}|\right|}.$$ (11)

Finally, we update the inter-group logits based on the agreement between the group capsule and the final output capsule:

$$b_{gj}^{\prime }=b_{gj}^{\prime }+{𝐯}_{j}^{g}\cdot {𝐯}_{j}^{final}.$$ (12)

This inter-group routing process iteratively refines the contribution of each group to the final capsule representation, ensuring that the most relevant features are retained in the final output. We propose the Divide-and-Conquer Routing Algorithm, as shown in Algorithm 2. The core idea of this approach is to partition the dynamic routing process into multiple groups, where each group focuses on learning distinct feature types. This partitioning improves the initialization of coupling coefficients and enhances the capture of key features. In our method, intra-group routing is first applied within each group to aggregate the capsules corresponding to specific, crucial features. Subsequently, inter-group routing is performed to combine the results from all groups into the final discriminative capsules.

**Algorithm 2 Divide-and-conquer routing algorithm.**

**Require:** Input capsules ${𝐮}_{i}$, number of intra-group routing iterations *r*_*intra*_, number of inter-group routing iterations *r*_*inter*_, number of groups *G*

**Ensure:** Output capsules ${𝐯}_{j}^{final}$

1: Divide the low-level capsules into *G* groups, where each group *g* contains $\frac{N}{G}$ capsules

2: // Intra-group routing initialization

3: **for** each group g∈G **do**

4:   Initialize the logits $b_{ij}^{g}=0$ for all capsules *i* in group *g* and all high-level capsules *j*

5: **end for**

6: // Intra-group iterative routing

7: **for** each group g∈G **do**

8:   **for** *r* = 1 to *r*_*intra*_ **do**

9:     Compute coupling coefficients using Coefficient Reverse Initialization using Eq 5:

10:    $c_{ij}^{g}=\frac{\exp(b_{ij}^{g})}{\sum_{k=1}^{\frac{N}{G}}\exp(b_{kj}^{g})}$

11:    Compute weighted sum of prediction vectors using Eq 6:

12:    ${𝐬}_{j}^{g}=\sum_{i\in g}c_{ij}^{g}{\hat{𝐮}}_{j|i}^{g}$

13:    Apply squashing function using Eq 7:

14:    ${𝐯}_{j}^{g}=\frac{||{𝐬}_{j}^{g}|{|}^{2}}{1+||{𝐬}_{j}^{g}|{|}^{2}}\frac{{𝐬}_{j}^{g}}{\left||{𝐬}_{j}^{g}|\right|}$

15:    Update logits based on agreement using Eq 8:

16:    $b_{ij}^{g}=b_{ij}^{g}+{\hat{𝐮}}_{j|i}^{g}\cdot {𝐯}_{j}^{g}$

17:   **end for**

18: **end for**

19: // Inter-group routing initialization

20: Initialize the inter-group logits $b_{gj}^{\prime }=0$ for all groups *g* and all high-level capsules *j*

21: // Inter-group iterative routing

22: **for** *r* = 1 to *r*_*inter*_ **do**

23:   Compute inter-group coupling coefficients using Eq 9:

24:   $c_{gj}^{\prime }=\frac{\exp(b_{gj}^{\prime })}{\sum_{k=1}^{G}\exp(b_{kj}^{\prime })}$

25:   Compute final high-level capsules using Eq 10:

26:   ${𝐬}_{j}^{final}=\sum_{g=1}^{G}c_{gj}^{\prime }{𝐯}_{j}^{g}$

27:   Apply squashing function using Eq 11:

28:   ${𝐯}_{j}^{final}=\frac{||{𝐬}_{j}^{final}|{|}^{2}}{1+||{𝐬}_{j}^{final}|{|}^{2}}\frac{{𝐬}_{j}^{final}}{\left||{𝐬}_{j}^{final}|\right|}$

29:   Update inter-group logits using Eq 12:

30:   $b_{gj}^{\prime }=b_{gj}^{\prime }+{𝐯}_{j}^{g}\cdot {𝐯}_{j}^{final}$

31: **end for**

32: **return** ${𝐯}_{j}^{final}$

### 3.4 Loss function

To optimize the training process within the framework of the divide-and-conquer routing algorithm, we introduce a margin loss function designed to encourage the model to learn discriminative features by promoting well-separated class representations. This loss function is constructed to leverage the enhanced capsule representations learned through intra-group and inter-group routing. The margin loss is defined as:

$$L_{c}=T_{c}\max(0,m^{+}-\|{𝐯}_{c}^{final}\|{)}^{2}+\lambda (1-T_{c})\max(0,\|{𝐯}_{c}^{final}\|-m^{-}{)}^{2},$$ (13)

where *T*_*c*_ is the target label for class *c*, *m*^ + ^ and *m*^−^ are the margin thresholds for the positive and negative classes, respectively, λ is a scaling factor, and ${𝐯}_{c}^{final}$ represents the final discriminative capsule output after inter-group routing. This loss function penalizes incorrect predictions by encouraging the model to generate capsule representations that are maximally separated for each class, thereby enhancing the model’s discriminative power, particularly within the context of the divide-and-conquer routing process.

## 4 Results

### 4.1 Datasets and implementation

#### 4.1.1 Datasets.

We evaluate our proposed DC-CapsNet on several widely used datasets in the computer vision domain. The datasets used in our experiments include MNIST [43], F-MNIST [44], K-MNIST [45], SVHN [46], CIFAR-10 [47], SMALLNORB [48], AFFNIST [49], IDRID [50], and ISIC [51]. We compare the performance of our model against the original capsule network architecture across these datasets.

The datasets are described as follows:

- **MNIST** [43]: This is a well-known dataset of handwritten digits containing 60,000 training images and 10,000 test images, each of size 28×28, spanning 10 classes. We use this dataset to evaluate the basic performance of our architecture on relatively simple, low-resolution data.
- **F-MNIST** [44]: Fashion-MNIST consists of 60,000 training images and 10,000 test images, each of size 28×28, depicting various clothing items across 10 categories. This dataset serves as a more challenging task compared to MNIST, with ten distinct classes of fashion items.
- **K-MNIST** [45]: The Kuzushiji-MNIST dataset is a collection of 70,000 handwritten Japanese characters, with 60,000 training images and 10,000 test images, each of size 28×28, covering 10 character classes. We use K-MNIST to test our model’s ability to handle more complex and diverse handwritten characters.
- **SVHN** [46]: The Street View House Numbers dataset consists of over 600,000 labeled digit images from real-world street scenes, with 10 classes (digits 0–9). The dataset provides a challenge due to its noisy and cluttered nature. The images are resized to 32×32×3 for our experiments.
- **CIFAR-10** [47]: CIFAR-10 consists of 60,000 images across 10 classes, each with 6,000 images of size 32×32×3. This dataset is widely used for evaluating models on general object recognition tasks.
- **SMALLNORB** [48]: SMALLNORB is a dataset of 50,000 3D rendered images of objects under various lighting conditions and viewpoints, covering 5 object categories with 10 instances each. The images are resized to 32×32×3 and used to assess our model’s ability to handle 3D object recognition and viewpoint variance.
- **AFFNIST** [49]: The AFFNIST dataset is a variant of MNIST in which digits are placed randomly on a black background of 40×40 pixels, with 10 classes and 35,000 test images. We train our model on the original MNIST dataset and test it on AFFNIST to assess its performance on more complex input layouts.
- **IDRID** [50]: The Indian Diabetic Retinopathy Image Dataset contains 516 retinal fundus images for diabetic retinopathy grading, annotated at the pixel level, and includes 3 disease categories. It is used to evaluate the model’s ability to handle medical image classification.
- **ISIC** [51]: The International Skin Imaging Collaboration dataset is a large collection of dermoscopic images for skin lesion analysis, including melanoma detection. The dataset contains 2,150 images with expert-provided labels across 3 classes and is used to assess the model’s performance on challenging medical image classification problems.

For MNIST, F-MNIST, K-MNIST, SMALLNORB, and AFFNIST, all images are resized to 28×28 before being fed into the network. For CIFAR-10, SVHN, IDRID, and ISIC, the images are resized to 32×32×3, and a random pixel shift of up to 2 pixels in each direction is applied using zero padding. No additional data augmentation or geometric transformations are applied to these datasets. For the AFFNIST dataset, the model is trained on MNIST with digits placed randomly on a black 40×40 pixel background and then tested on the AFFNIST dataset. For all other datasets, the original image sizes are used as provided.

#### 4.1.2 Experimental setup.

Our experiments are implemented using the PyTorch library, which provides a flexible framework for training and evaluating deep learning models. The training procedure utilizes the Adam optimizer [52] with an initial learning rate of 0.0001, which is reduced by 5% after every epoch. This learning rate schedule helps the model converge more efficiently while mitigating overfitting. The batch size is set to 128, meaning the model is trained with 128 images per iteration. Each experiment is trained for 300 epochs, ensuring sufficient training time for the model to converge across all datasets. To ensure robustness, all experiments are run three times, and the results are averaged to account for variability in training. This approach helps mitigate randomness introduced during training and provides a more reliable estimate of model performance.

### 4.2 Performance evaluation

#### 4.2.1 Comparison with dynamic routing algorithm.

Table 1 presents a comprehensive comparison of classification accuracy (%) and standard deviation across multiple image datasets for CNN, CapsNet-DR, CapsNet-CRI, DC-CapsNet-DR, and our proposed DC-CapsNet. The inclusion of a CNN baseline with a comparable parameter size provides a reference for conventional convolutional architectures. The results show that CapsNet-DR generally outperforms both CNN and CapsNet-CRI, confirming the effectiveness of dynamic routing. However, CapsNet-CRI, which uses inverse initialization of coupling coefficients, suffers a noticeable drop in performance and higher variance, indicating that excessively small initialization values hinder the contribution of lower-level capsules. Notably, DC-CapsNet-DR, which applies the divide-and-conquer grouping strategy directly to dynamic routing, also achieves improved performance over CapsNet-DR on most datasets, demonstrating that the group-wise routing structure itself brings efficiency and robustness benefits. However, its performance is still inferior to our full DC-CapsNet method, which further incorporates coefficient reverse initialization. This gap highlights that simply grouping capsules is not sufficient; addressing the inconsistency between coupling coefficient initialization and the capsule aggregation direction is crucial. Our proposed divide-and-conquer routing, which aligns initialization with the aggregation structure, is therefore necessary for optimal performance. DC-CapsNet achieves the best or competitive accuracy on most datasets, especially on general image classification and medical tasks, and consistently exhibits lower standard deviation, demonstrating improved robustness and stability.

It is important to note, however, that on datasets specifically designed to evaluate invariance to spatial transformations, such as SMALLNORB and AFFNIST, DC-CapsNet does not outperform the original dynamic routing method. The performance gap is particularly evident on SMALLNORB, where the accuracy of DC-CapsNet is more than 2% lower. This result highlights a key limitation of our approach. While the divide-and-conquer strategy improves local feature discrimination and computational efficiency, it may reduce the model’s ability to capture global spatial relationships and complex pose variations, which are critical for pose-invariant modeling. By partitioning the routing process into localized feature subspaces, the grouping mechanism may unintentionally weaken the global agreement needed to represent holistic object transformations. This trade-off suggests that the proposed method is particularly suitable for tasks that prioritize local discriminative features, such as medical image analysis and general classification benchmarks, but may be less effective in scenarios requiring strong modeling of global spatial dependencies and viewpoint changes.

**Fig 3:**
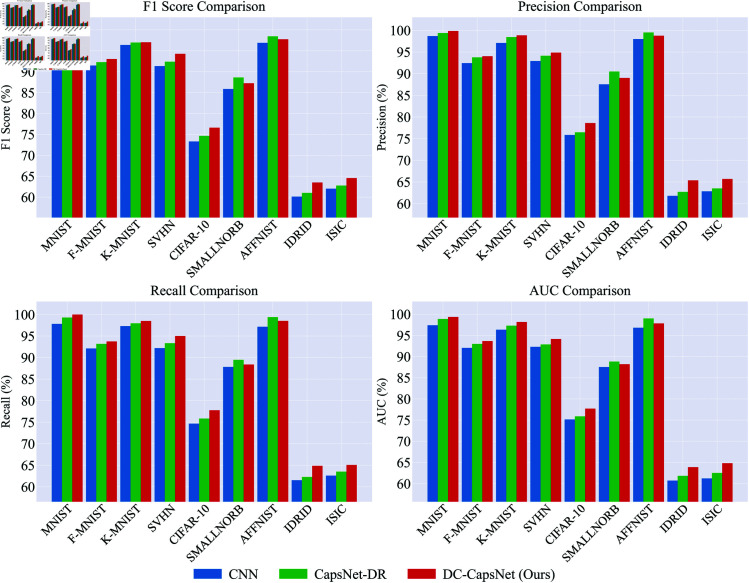
Comparison of F1 score, Precision, Recall, AUC among CNN, CapsNet-DR and DC-CapsNet across different datasets.

Fig 3 presents a comparative evaluation of CNN, CapsNet-DR, and DC-CapsNet across multiple datasets in terms of F1 score, Precision, Recall, and AUC. As illustrated in the figure, DC-CapsNet consistently outperforms the original dynamic routing method on most datasets and metrics, with particularly notable improvements in comprehensive evaluation indicators such as F1 score and AUC. Notably, CNN exhibits the lowest scores across all metrics compared to both capsule network routing algorithms, further highlighting the superiority of capsule-based approaches in capturing complex feature relationships. These results validate the effectiveness of our proposed divide-and-conquer routing strategy, which not only enhances classification accuracy but also improves model robustness and generalization across diverse tasks. The superior performance demonstrates the method’s capability to capture and differentiate salient features, particularly in datasets with complex patterns or medical imaging tasks, where precise feature representation is critical for accurate classification.

Table 2 provides a detailed comparison of runtime (seconds per epoch), parameter count (in millions), and memory consumption (MB) between DC-CapsNet and CapsNet-DR on the MNIST and SVHN datasets. These two datasets are selected as representatives due to their differing image resolutions, which are indicative of the broader range of datasets evaluated. The observed trends in runtime, parameter efficiency, and memory usage are consistent across all datasets considered in this study. As shown in Table 2, DC-CapsNet achieves a substantial reduction in computational cost, with lower runtime per epoch and decreased memory consumption compared to CapsNet-DR. Specifically, DC-CapsNet reduces the number of parameters by nearly half, which directly translates to improved memory efficiency and faster training. The reduction in runtime and memory footprint is particularly significant for larger or higher-resolution datasets, where computational resources are a critical consideration. These results demonstrate that the proposed divide-and-conquer routing strategy not only enhances classification performance but also offers practical advantages in terms of scalability and resource utilization, making it well-suited for deployment in real-world scenarios with limited computational budgets.

**Table 2:** Comparison of runtime (seconds per epoch), number of parameters (M), and memory consumption (MB) for CapsNet-DR and DC-CapsNet (Ours) on different datasets.

Model	Runtime (s/epoch)		Parameters (M)		Memory (MB)	
Dataset	CapsNet-DR	DC-CapsNet	CapsNet-DR	DC-CapsNet	CapsNet-DR	DC-CapsNet
MNIST	21.10	**17.21**	5.33	**2.68**	2503	**1402**
SVHN	43.17	**32.46**	5.37	**2.72**	3874	**2170**

In summary, the experimental results comprehensively demonstrate the superiority of the proposed DC-CapsNet over both conventional CNNs and existing capsule network variants. Quantitative comparisons in Table 1 reveal that DC-CapsNet consistently achieves higher or comparable classification accuracy with reduced variance across a diverse set of image classification and medical imaging benchmarks. The ablation with CapsNet-CRI further highlights the importance of appropriate coupling coefficient initialization, as excessively small values can impede effective feature aggregation. Qualitative metrics presented in Fig 3 reinforce these findings, with DC-CapsNet exhibiting notable improvements in F1 score, Precision, Recall, and AUC, thereby underscoring its enhanced robustness and generalization capability. Furthermore, the efficiency analysis in Table 2 demonstrates that the divide-and-conquer routing strategy substantially reduces computational overhead, parameter count, and memory consumption without compromising predictive performance. Collectively, these results validate the effectiveness and practicality of DC-CapsNet, establishing it as a scalable and robust solution for complex visual recognition tasks in resource-constrained environments.

#### 4.2.2 Improvements combined with advanced routing algorithms.

The experimental results presented in Table 3 demonstrate the performance improvements achieved by integrating the proposed divide-and-conquer routing algorithm into various advanced capsule network routing methods, including AR-CapsNet [31], AA-Caps [36], RS-CapsNet [42], FR-CapsNet [53], IAR-CapsNet [37], and GR-CapsNet [32]. To ensure a fair comparison, we implement these advanced routing methods following the experimental configurations outlined in [54, 55]. Specifically, our approach consistently enhances classification accuracy across all datasets.

**Table 3:** Experimental results comparing the classification accuracy (Acc., %) and standard deviation (Std.) of advanced capsule network routing algorithms with the proposed DC-CapsNet method. The values in parentheses represent the standard deviation of three independent runs.

Model	Metric	MNIST		F-MNIST		CIFAR-10		SVHN		IDRID		ISIC	
		Baseline	Ours	Baseline	Ours	Baseline	Ours	Baseline	Ours	Baseline	Ours	Baseline	Ours
AR-CapsNet [31]	Acc.	99.46	**99.52**	92.98	**93.15**	87.19	**87.42**	92.47	**93.07**	61.58	**64.21**	62.87	**65.12**
	Std.	±0.00021	±0.00013	±0.00047	±0.00029	±0.00185	±0.00112	±0.00038	±0.00022	±0.00019	±0.00011	±0.00310	±0.00210
AA-Caps [36]	Acc.	99.50	**99.55**	93.02	**93.23**	71.60	**72.35**	92.13	**92.32**	61.72	**64.35**	62.95	**65.20**
	Std.	±0.00019	±0.00012	±0.00044	±0.00027	±0.00192	±0.00108	±0.00035	±0.00020	±0.00018	±0.00010	±0.00300	±0.00205
RS-CapsNet [42]	Acc.	99.54	**99.61**	93.51	**93.85**	89.81	**90.17**	96.50	**96.72**	61.85	**64.48**	63.10	**65.28**
	Std.	±0.00018	±0.00011	±0.00042	±0.00025	±0.00188	±0.00105	±0.00032	±0.00018	±0.00017	±0.00009	±0.00295	±0.00200
FR-CapsNet [53]	Acc.	99.55	**99.63**	93.62	**94.14**	90.23	**90.62**	96.57	**96.79**	61.92	**64.52**	63.18	**65.35**
	Std.	±0.00017	±0.00010	±0.00041	±0.00023	±0.00185	±0.00102	±0.00030	±0.00017	±0.00016	±0.00009	±0.00290	±0.00198
IAR-CapsNet [37]	Acc.	99.58	**99.65**	93.83	**94.52**	90.62	**91.14**	96.62	**96.82**	62.05	**64.65**	63.25	**65.40**
	Std.	±0.00016	±0.00009	±0.00039	±0.00021	±0.00180	±0.00100	±0.00028	±0.00016	±0.00015	±0.00008	±0.00285	±0.00195
GR-CapsNet [32]	Acc.	99.61	**99.67**	93.85	**94.55**	90.67	**91.25**	96.65	**96.87**	62.10	**64.72**	63.30	**65.42**
	Std.	±0.00015	±0.00008	±0.00038	±0.00020	±0.00178	±0.00098	±0.00027	±0.00015	±0.00014	±0.00008	±0.00280	±0.00192

For each baseline method (AR-CapsNet, AA-Caps, RS-CapsNet, FR-CapsNet, IAR-CapsNet, and GR-CapsNet), we replace the original fully connected routing module with our group-based divide-and-conquer routing framework, while preserving the method-specific update rules and attention mechanisms within each group. Intra-group routing follows the protocol of each respective method, such as attention-based routing for AR-CapsNet and self-attention for AA-Caps. After intra-group updates, an inter-group routing step aggregates the intermediate outputs, ensuring that while the core routing logic of each method is retained, the overall routing structure benefits from reduced complexity and improved coefficient initialization as enabled by DC-CapsNet. All other training configurations and hyperparameters are kept identical to those in the original implementations to ensure a fair comparison. This modular integration strategy allows our framework to enhance both feature discriminability and generalization across a wide range of capsule routing paradigms, as reflected in the consistent accuracy gains and reduced performance variance reported in Table 3. The improvements indicate that our method effectively strengthens feature representation learning in capsule networks, allowing for more robust and discriminative feature extraction. Notably, the enhancement is more pronounced in challenging datasets such as CIFAR-10, IDRID, and ISIC, where capturing fine-grained features is crucial. The reduction in standard deviation further indicates enhanced robustness and stability of the proposed method. These findings validate the effectiveness of our method in optimizing dynamic routing mechanisms, leading to superior classification performance in various image recognition benchmarks.

**Fig 4:**
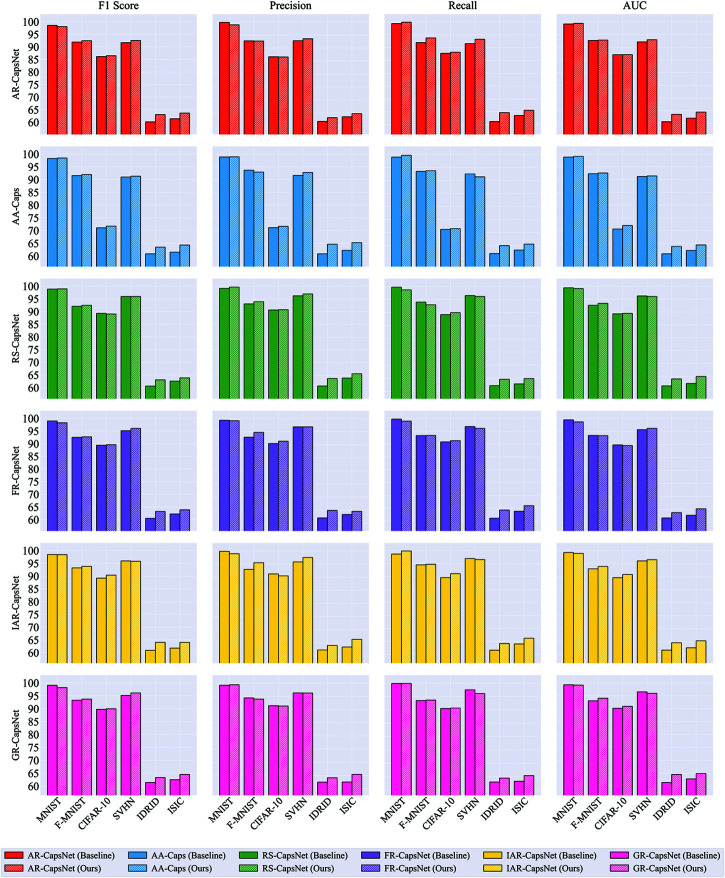
Comparison of F1 score, Precision, Recall, and AUC for different advanced capsule network routing algorithms (baseline) and their improvements with our method across various datasets.

As illustrated in Fig 4, our method consistently achieves improvements over the baseline routing algorithms in terms of F1 score, Precision, Recall, and AUC across all datasets and experimental settings. In every case, integrating the divide-and-conquer routing strategy leads to superior performance, highlighting its effectiveness in enhancing both the discriminative power and generalization ability of capsule networks.

In summary, the results presented in Table 3 and Fig 4 demonstrate that the proposed divide-and-conquer routing algorithm consistently enhances the performance of advanced capsule network routing methods. It achieves improvements in both accuracy and a range of comprehensive evaluation metrics, including F1 score, Precision, Recall, and AUC, across multiple datasets. These performance gains reflect not only stronger feature representation learning but also increased robustness and stability. Overall, the findings highlight the practical significance and broad applicability of the proposed approach for advancing capsule-based architectures in challenging visual recognition and medical imaging scenarios.

### 4.3 Ablation studies

#### 4.3.1 Effect of group size.

To investigate the impact of group size on model performance, we conducted an ablation study by varying the number of groups while keeping the total number of low-level capsules constant. Specifically, we evaluated models with group sizes of 16, 32, and 64, and reported the corresponding classification accuracy and standard deviation across six datasets, including MNIST, F-MNIST, CIFAR-10, SVHN, IDRID, and ISIC, as summarized in Table 4.

**Table 4:** Experimental results evaluating the impact of different group numbers in the proposed DC-CapsNet. The results demonstrate how varying the number of groups affects classification accuracy (Acc., %) and standard deviation (Std.) across different datasets.

Group Size	Metric	MNIST	F-MNIST	CIFAR-10	SVHN	IDRID	ISIC
16	Acc.	99.61	93.05	75.93	93.52	63.82	64.35
	Std.	±0.00018	±0.00044	±0.00192	±0.00033	±0.00015	±0.00300
32	Acc.	**99.67**	**93.13**	**76.17**	**93.81**	**64.75**	**65.45**
	Std.	±0.00012	±0.00029	±0.00128	±0.00022	±0.00010	±0.00200
64	Acc.	99.65	93.11	76.04	93.67	64.12	64.92
	Std.	±0.00016	±0.00031	±0.00135	±0.00025	±0.00012	±0.00210

As shown in Table 4, the model achieves the highest accuracy and lowest standard deviation across all datasets when the group size is set to 32. In contrast, both smaller (16) and larger (64) group sizes result in a slight decrease in accuracy and increased performance variability, with a group size of 64 generally outperforming a group size of 16. Notably, the improvements are consistent not only on standard benchmarks such as MNIST and CIFAR-10, but also on challenging medical imaging datasets like IDRID and ISIC, where the optimal group size of 32 yields the most robust and stable results. These findings suggest that a group size of 32 provides the best trade-off between computational efficiency and model expressiveness, enabling the network to capture a diverse set of feature representations without incurring unnecessary computational overhead or sacrificing generalization. This observation holds across both natural and medical image domains, further validating the effectiveness and generalizability of the proposed divide-and-conquer routing strategy.

#### 4.3.2 Effect of routing iterations.

To evaluate the impact of routing iterations on model performance, we conducted an ablation study on different datasets by varying the number of intra-group and inter-group routing iterations while keeping other hyperparameters constant. The classification accuracy of DC-CapsNet was assessed under routing iteration settings of 1, 3, and 5. The experimental results, including the standard deviation across three independent runs, are presented in Table 5 for six representative datasets: MNIST, F-MNIST, CIFAR-10, SVHN, IDRID, and ISIC.

**Table 5:** Evaluation of the impact of different routing iterations on the classification accuracy (Acc., %) and standard deviation (Std.) of the proposed DC-CapsNet across various datasets. The results illustrate how variations in the number of routing iterations affect model performance.

Intra-Group	Inter-Group	Metric	MNIST	F-MNIST	CIFAR-10	SVHN	IDRID	ISIC
1	1	Acc.	99.60	93.01	75.84	93.64	63.70	64.30
		Std.	±0.00018	±0.00044	±0.00192	±0.00033	±0.00015	±0.00300
1	3	Acc.	99.65	93.10	76.08	93.78	64.50	65.20
		Std.	±0.00012	±0.00029	±0.00128	±0.00022	±0.00010	±0.00200
1	5	Acc.	99.63	93.06	75.97	93.72	64.20	64.90
		Std.	±0.00016	±0.00031	±0.00135	±0.00025	±0.00012	±0.00210
3	1	Acc.	99.62	93.04	75.91	93.68	63.95	64.60
		Std.	±0.00018	±0.00044	±0.00192	±0.00033	±0.00015	±0.00300
3	3	Acc.	**99.67**	**93.13**	**76.17**	**93.81**	**64.75**	**65.45**
		Std.	±0.00012	±0.00029	±0.00128	±0.00022	±0.00010	±0.00200
3	5	Acc.	99.64	93.08	76.02	93.75	64.30	65.10
		Std.	±0.00016	±0.00031	±0.00135	±0.00025	±0.00012	±0.00210
5	1	Acc.	99.61	93.02	75.89	93.70	63.82	64.35
		Std.	±0.00018	±0.00044	±0.00192	±0.00033	±0.00015	±0.00300
5	3	Acc.	99.63	93.07	76.03	93.76	64.40	65.18
		Std.	±0.00012	±0.00029	±0.00128	±0.00022	±0.00010	±0.00200
5	5	Acc.	99.58	92.98	75.79	93.60	63.70	64.28
		Std.	±0.00016	±0.00031	±0.00135	±0.00025	±0.00012	±0.00210

As shown in Table 5, DC-CapsNet achieves the highest accuracy and lowest standard deviation when both intra-group and inter-group routing iterations are set to 3, consistently across all evaluated datasets. Notably, this trend holds for both standard benchmarks (MNIST, CIFAR-10) and challenging medical imaging datasets (IDRID, ISIC), demonstrating the robustness and generalizability of the proposed routing strategy. Increasing the number of routing iterations beyond three does not yield further improvements in accuracy and, in some cases, leads to marginal increases in variance, suggesting diminishing returns and unnecessary computational overhead. Conversely, using only a single routing iteration results in suboptimal performance and higher variability, indicating insufficient feature refinement. These findings confirm that three routing iterations provide an optimal trade-off between convergence efficiency, classification accuracy, and model stability, making this configuration well-suited for a wide range of visual recognition tasks, including those in the medical domain.

### 4.4 Visualization

#### 4.4.1 Visualization of group-wise capsule representations.

**Fig 5:**
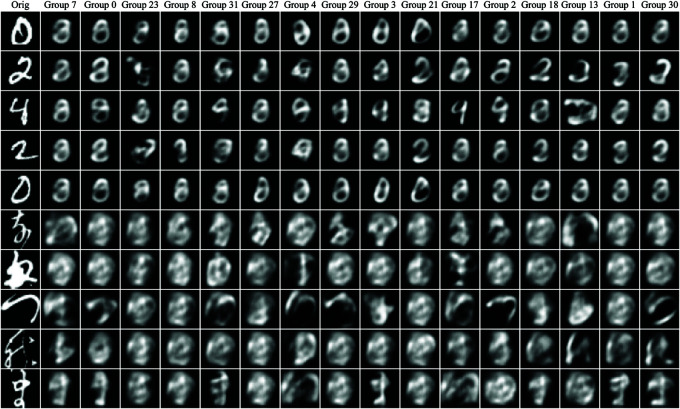
Comparison of the original images and visualizations from different capsule groups. The first five rows display samples from the MNIST dataset, while the last five rows are from the KMNIST dataset. The **Orig** column shows the original input image, and the **Group + number** columns present reconstructed features derived from different capsule groups. These visualizations demonstrate how distinct capsule groups capture specific image features, offering clearer and more focused representations than the original input. This improved interpretability contributes to better discrimination and enhanced performance in downstream tasks.

To better understand the features learned by different capsule groups, we visualize and compare the original input images with the outputs of selected capsule groups. As shown in Fig 5, we randomly sample 12 capsule groups and display their corresponding activations. Each group captures distinct local representations from the input, highlighting specific visual attributes such as edges, textures, and shapes. These visualizations reveal how different capsule groups specialize in distinct aspects of the input, thereby enhancing the model’s representational diversity and discriminative capacity. The results underscore the effectiveness of the proposed divide-and-conquer routing strategy in learning diverse, interpretable feature representations that contribute to improved performance and explainability.

#### 4.4.2 Reconstruction comparison with dynamic routing.

**Fig 6:**
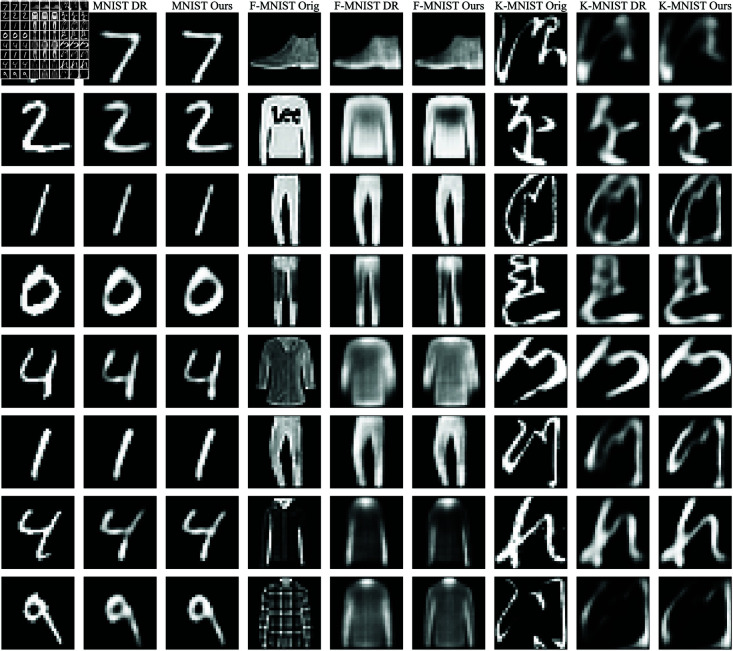
Comparison of input image reconstruction results between the dynamic routing algorithm and our method on the MNIST, F-MNIST, and K-MNIST datasets. The **MNIST+Orig**, **F-MNIST+Orig**, and **K-MNIST+Orig** columns represent the original images from the respective datasets, while the **MNIST+DR**, **F-MNIST+DR**, and **K-MNIST+DR** columns show the reconstructed images using the dynamic routing algorithm. The **MNIST+Ours**, **F-MNIST+Ours**, and **K-MNIST+Ours** columns display the reconstructed images using our proposed method.

To further evaluate the representational quality of different routing strategies, we compare reconstruction results produced by DC-CapsNet and the original dynamic routing algorithm across multiple datasets. As illustrated in Fig 6, our method consistently generates sharper and more detailed reconstructions, with fewer blurred or distorted regions compared to the dynamic routing algorithm. This improvement demonstrates that the proposed divide-and-conquer routing strategy enables the network to capture more structured and informative latent features. The ability to reconstruct fine-grained details from the capsule representations indicates stronger feature preservation and better encoding of spatial relationships. Overall, these findings highlight the superiority of DC-CapsNet in retaining essential visual information, which contributes to both improved interpretability and downstream task performance.

## 5 Discussion

Our experimental results provide compelling evidence of the advantages offered by the proposed DC-CapsNet architecture over the original CapsNet. The observed improvements across all datasets strongly suggest that the divide-and-conquer strategy significantly enhances the model’s capacity to learn discriminative features, particularly in complex scenarios. The success of the proposed approach highlights the potential of partitioning the routing process into smaller groups, thereby reducing computational overhead while maintaining or even improving performance, especially when dealing with datasets exhibiting high feature variability.

### 5.1 Impact of the divide-and-conquer strategy

The divide-and-conquer strategy at the core of DC-CapsNet significantly contributes to the model’s success. By partitioning the routing process into smaller groups that focus on distinct aspects of the feature space, the model learns more focused and discriminative representations. This segmentation not only reduces computational overhead but also ensures that the model effectively handles diverse datasets. The improvements observed in datasets with high variability further validate that DC-CapsNet generalizes better by capturing distinct feature representations within each group. This capability suggests that DC-CapsNet could be particularly advantageous for applications involving large and complex datasets, where traditional approaches often struggle.

### 5.2 Generalization across diverse datasets

One of the most significant findings from our experiments is DC-CapsNet’s ability to effectively generalize across different datasets. The method is particularly adept at capturing subtle variations and patterns within images, which enhances its adaptability to a wide range of visual recognition tasks. Whether working with simple or more complex datasets, DC-CapsNet demonstrates that its approach to dividing the feature space into smaller groups enables more robust feature learning. This ability to adapt to diverse datasets, especially those with high feature diversity or noise, underscores the robustness of the proposed method. DC-CapsNet’s group-wise routing process facilitates better generalization by allowing the model to focus on smaller subsets of the feature space, leading to improved performance in scenarios where traditional models may struggle to capture the underlying patterns effectively.

### 5.3 Limitations in capturing complex spatial relationships

While DC-CapsNet demonstrates strong performance across most datasets, there are some limitations when it comes to modeling complex spatial relationships and transformations. As shown in Table 1, DC-CapsNet achieves slightly lower accuracy on the **SMALLNORB** dataset compared to the original CapsNet. This dataset is particularly challenging due to its emphasis on diverse object viewpoints and spatial configurations. The divide-and-conquer strategy, by grouping capsules, may reduce the ability to aggregate global spatial information that is important for such tasks. However, it is important to note that even in these challenging scenarios, DC-CapsNet consistently outperforms a CNN baseline with a comparable number of parameters across classification evaluation metrics, including accuracy and standard deviation. This indicates that DC-CapsNet maintains robust feature learning and generalization ability, even when its accuracy is marginally lower than that of the original CapsNet. Future work could focus on enhancing the inter-group interactions to better capture global spatial dependencies, further improving performance on tasks requiring complex spatial reasoning.

### 5.4 Optimizing group partitioning for scalability

Further optimization of the group partitioning strategy could improve scalability and efficiency. Currently, the number of groups is predefined, which may not be optimal for every dataset. In practice, datasets with varying complexity may benefit from dynamic or data-driven group allocation methods, which could adjust the number of groups based on feature complexity or the difficulty of the learning task. By adopting more flexible group partitioning strategies, we could potentially reduce training time while maintaining or even improving classification performance. Such dynamic allocation would enable DC-CapsNet to better scale across datasets of different sizes and complexities, making it more versatile for real-world applications.

### 5.5 Hardware-aware optimization for real-world applications

Another avenue for improving the efficiency of DC-CapsNet lies in hardware-aware optimization. Tailoring the routing operations to GPU or TPU architectures would not only accelerate the training process but also make DC-CapsNet more practical for deployment in resource-constrained environments. Such hardware-aware implementations could enhance both the speed and practicality of DC-CapsNet, making it a viable solution for large-scale, real-world applications. Further exploration of parallelized routing methods and optimizations tailored to specific hardware could yield additional efficiency gains, helping the model achieve faster convergence and broader applicability in real-time systems.

## 6 Conclusion

In this study, we propose DC-CapsNet, which enhances the original dynamic routing algorithm by introducing a divide-and-conquer strategy. This approach improves the initialization of coupling coefficients and strengthens the recognition of critical local features. By partitioning the routing process into smaller groups, DC-CapsNet effectively focuses on different feature types while reducing computational complexity. Experimental results on multiple image classification datasets demonstrate that DC-CapsNet achieves superior classification accuracy compared to the original CapsNet. Furthermore, the proposed strategy integrates seamlessly with advanced routing algorithms, further enhancing feature discrimination and generalization across various computer vision tasks.

In future work, we plan to explore adaptive group partitioning strategies that dynamically adjust group sizes based on the complexity of the data. Additionally, we will investigate methods to enhance the inter-group communication for improved global feature modeling. Extending DC-CapsNet to large-scale and real-time applications is also a promising direction.
